# The Extent of the Preserved Feathers on the Four-Winged Dinosaur *Microraptor gui* under Ultraviolet Light

**DOI:** 10.1371/journal.pone.0009223

**Published:** 2010-02-15

**Authors:** David W. E. Hone, Helmut Tischlinger, Xing Xu, Fucheng Zhang

**Affiliations:** 1 Key Laboratory of Evolutionary Systematics of Vertebrates, Institute of Vertebrate Paleontology and Paleoanthropology, Chinese Academy of Sciences, Beijing, China; 2 Tannenweg 16, D-85134, Stammham, Germany; Raymond M. Alf Museum of Paleontology, United States of America

## Abstract

**Background:**

The holotype of the theropod non-avian dinosaur *Microraptor gui* from the Early Cretaceous of China shows extensive preservation of feathers in a halo around the body and with flight feathers associated with both the fore and hindlimbs. It has been questioned as to whether or not the feathers did extend into the halo to reach the body, or had disassociated and moved before preservation. This taxon has important implications for the origin of flight in birds and the possibility of a four-winged gliding phase.

**Methodology/Principal Findings:**

Examination of the specimen under ultraviolet light reveals that these feathers actually reach the body of the animal and were not disassociated from the bones. Instead they may have been chemically altered by the body tissues of the animal meaning that they did not carbonise close into the animal or more likely were covered by other decaying tissue, though evidence of their presence remains.

**Conclusions/Significance:**

These UV images show that the feathers preserved on the slab are genuinely associated with the skeleton and that their arrangement and orientation is likely correct. The methods used here to reveal hidden features of the specimen may be applicable to other specimens from the fossil beds of Liaoning that produced *Microraptor*.

## Introduction

### UV Light

Most skeletal remains of fossil bones and shells and sometimes also mineralized soft parts from different Upper Jurassic plattenkalks of Southern Franconia in Germany (commonly known as “Solnhofen-Fossils”) are fluorescent under ultraviolet radiation (e.g. see 1). First examinations of Solnhofen fossils under ultraviolet light, including vertebrate skeletal remains and some crabs, were carried out as early as in 1928 but during the ensuing decades, primarily invertebrates (with a focus on crustaceans) were documented. Under the available low powered lights and with basic investigation and photographic techniques only very brightly fluorescing bones and other hard parts were visible. However, in the last 10 years especially, ultraviolet investigation techniques and ultraviolet-light photography have been improved considerably allowing documentation of Solnhofen fossils including dinosaurs and a number of *Archaeopteryx* specimens [1]–[5]. New details of both skeletal material and soft tissues including feathers [e.g. 5] can be studied in greater detail than before, and new observations made based on additional information.

It is now clear that in the majority of cases morphological details of skeletal remains as well as the remains of soft parts can be more precisely examined in ultraviolet light than in visible light at the macro scale (as opposed to, say, examination under an SEM). Often delicate elements, including different bony components and remains of soft parts, are poorly discernable or cannot be seen in visible light but fluoresce conspicuously under filtered UV [e.g. 6]. The technique can be used to show otherwise hidden bony sutures, and to separate bones or soft parts from the underlying matrix or each other. Plaster and glue (e.g. polyester or epoxy) as well as restored parts and other artifacts made by synthetic material also fluoresce brightly. Our preliminary investigations here suggest that, in common with the Solnhofen limestones, the Mesozoic fossil beds of exceptional preservation in northern China – the Yixian (shown here) and Daohugou formations [see 7] can also reveal many new morphological details through the use of UV lighting.

### Fossil Feathers in China

The fossil beds of Liaoning province in northeastern China have revealed a great deal of information about feathered non-avian theropods as well as basal avialans [8] from both the Daohuguo and Tiaojishan formations of the Late Jurassic and the Jehol group of the Early Cretaceous [9]. With oviraptorosaurs [e.g. *Caudipteryx* – 10], dromaeosaurs [e.g. *Microraptor –* 11], troodontids [e.g. *Anchiornis* ]–[9, 12], basal avialans [e.g. *Pedopenna* – 13], and early birds [e.g. *Jeholornis* – 14] all being represented by specimens preserving derived avian-like feathers, much new information about the possible origin and the evolution of avian flight has been gained [8], [15], [16].

Since its discovery, the small dromaeosaurid theropod *Microraptor gui* [11] has been the subject of intense interest [e.g. 17]–[20]. The presence of ‘wings’, or more specifically groups of asymmetrical feathers, on both the manus and pes of the animal suggest that it could generate lift with both fore and hind limbs [11]. This condition has led to the rejuvenation of the idea that avian flight may have evolved from such a four-winged planform [21] and has led to direct research into the aerodynamic capabilities of *Microraptor* [19] and the leg feathers of the famous basal avialan, the Late Jurassic *Archaeopteryx* [5], [22].

Longrich [22] asserted that the feather structure and arrangement in the hindlimbs of *Archeopteryx* indicated that they were used as lift-generating winglets, and calculated that these structures could have significantly decreased both the stall speed and turning radius of the bird. In contrast, Tischlinger and Unwin [4] highlighted that these feathers are equivalent to the “feather trousers” of many extant birds (e.g. birds of prey), that as in *Archaeopteryx* are exclusively attached to the tibia and femur. Tischlinger and Unwin concluded that the putative orientation of the feathers in one plane is a taphonomic artefact (as in some other fossil birds) and not as a result of this orientation being a natural one on the animal. Wellnhofer [23, p.135] asserted that the leg feathers of *Archaeopteryx*, being relatively short contour feathers, could not have functioned as an airfoil during flight. More recently, the discovery of a basal troodontid [9] with extensive feathers on the hindlimbs suggests that this may be a primitive condition for Aves. Since current phylogenies suggest that Paraves (that is, birds, dromaeosaurs and troodontids) are all closely related [e.g. 12], [15] and that both troodontids and dromaeosaurs are preserved with feathers on the metatarsus, the most parsimonious interpretation would suggest that the lack of feathers on the metatarsus of *Archaeopteryx* is derived.

**Figure 1:**
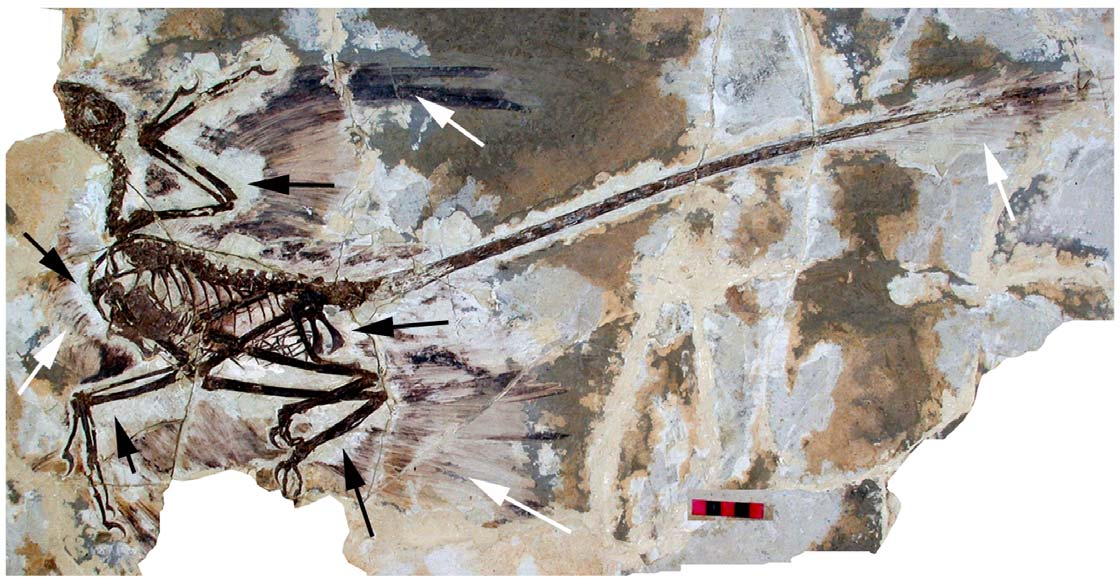
The holotype of *Microraptor gui*, IVPP V 13352 under normal light. This shows the preserved feathers (white arrow) and the ‘halo’ around the specimen where they appear to be absent (black arrows). Scale bar at 5 cm.

Xu et al. [11] described the wing feathers of *Microraptor gui* as being “preserved in a pattern similar to that of modern birds” (p 338) and that the “leg feathers are arranged in a pattern similar to the arm feathers”. On the holotype specimen there are long pennaceous feathers on the tibia but also longer feathers on the whole of the metatarsus with the longest feathers positioned distally. However, the exact orientation and association of the feathers in *Microraptor gui* with the limbs have been the subject of some discussion [e.g. 19], [20], [22], [24]. The feathers appear to be carbonised (a common feature of feathered specimens from China) and are preserved as a black carbon stain on the matrix [25]. However, in the region close to the bones of the specimen, the feathers are not visible leaving a ‘halo’ effect between the bones and the feathers (Figure 1). Notably, the proximal tibial feathers which were likely present cannot be seen as a result of this halo.

In extant birds, feathers are rooted deeply in the soft tissue of the animal with the basal part of the shaft (the calamus) residing in a follicle of the epidermis [26 Chapter 4]. They can be moved in most cases by small smooth muscles [26 Chapter 4] which provide further anchorage, and in many cases, the follicles are also attached to tendons or ligaments with the very base of the feather touching or articulating with a bone and in the wing [e.g. 27 Chapter 3]. As such, feathers can be considered to be strongly attached to the body of the animal, and are not easily disturbed. This implies that in many cases, the position of feathers as preserved on fossils will accurately reflect the position in life.

In many other Jehol group fossil specimens, feathers lie on the rock such that they meet or nearly meet the bones of the animal [e.g. see 12], [28], [29]. However, in the *Microraptor gui* holotype this pattern of feathers reaching bones is disrupted by the halo. Thus it is not clear if the feathers as seen are larger and do extend to the bones, or if they were perhaps dissociated and moved, perhaps by a current, before they settled and were preserved. This halo effect can be seen in other specimens as well [e.g. see 9], [30]. One would expect the proximal part of a feather to actually reach, if not articulate with major bones in the skeleton, as this occurs in modern birds [e.g. see 31] and osteological correlates of these attachments (‘quill knobs’) are known in the dromaeosaur *Velociraptor* [32].

It is therefore unclear quite how the feathers may have been associated with the bones of the skeleton of *Microraptor gui,* despite the interest in this taxon and the importance of this issue for both flight and terrestrial locomotion. In their original paper, Xu and colleagues [11] considered the feathers to be incompletely preserved in the holotype and made the perhaps safe assumption that the feathers were incompletely preserved, rather than having been moved on the slab. However, their measurements were based on the feathers as seen and did not include their putative extension into the halo. Similarly, Chatterjee and Templin [19] considered the longest metatarsal feathers to be 14 cm long, which precludes any extension into the halo. Padian [33] was more critical, noting that “there's insufficient evidence of the attachment of these feathers to the hind limbs…There seems to be a gap between the vaned area of feathers that are near the hind limbs and the bones of the hind limbs themselves”, a comment echoed by Padian and Dial [18].

Here we present views of the *M. gui* holotype under ultraviolet light showing the feathers articulating with the bones of the specimen as seen in other avian and non-avian dinosaur specimens and conclude that the feathers of the *Microraptor* holotype are therefore in a natural position.

## Methods

Specimen IVPP V 13352 (Institute of Vertebrate Paleontology and Paleoanthropology, Beijing, China), the holotype of *Microraptor gui* (11), was examined and photographed under UV light. Images were taken on slides (Kodak Professional Elitechrome ISO 100/21° and later scanned) and with a digital SLR.

UV-A lamps with a wavelength of 365–366 nanometers were used. Powerful modern UV-A lamps guarantee a UV intensity between 4 000 and more than 90 000 microwatts per cm^2^, depending on the distance concerned and the number of lamps. (Note that when unprotected eyes and skin are exposed to artificial UV-A of high intensity, the recommended T_max_ values of the manufacturers, usually between 5 and 30 minutes, must not be exceeded. In any case it is safer to cover skin of the hands and forearms with clothing and the eyes with UV-blocking glasses or goggles from the beginning of any UV investigation).

Sometimes essential details of bones and soft parts can exclusively be demonstrated by ultraviolet-light photography due to the fact that the researcher will not be able to differentiate tiny structures and differences in colour and composition under ultraviolet light with the naked eye or with a microscope. These differences are enhanced considerably by an established filtering technique which is crucial for photographic documentation. The application of different filters allows a selective visualisation of peculiar fine structures by providing additional contrast. Colour correction filters (yellows, blues and reds of different types and densities and in different combinations) are made from special coloured glass or polyester and are affixed to the camera or microscope lens. In most cases a selection of different colour correction filters is necessary. The first filter has to be a UV Filter which is supposed to block UV light up to 390 nanometers (e.g. Hama or Hoya brand O-Haze).

On each stone slab, bone or tissue will react differently to different light wavelengths and will be captured differently with varying exposures and filters - a blanket approach to formations or even horizons is not advised. This appears to be true even of plates and counterplates of single specimens in some cases, and not just more predictable differences between different horizons or formations of rock. Thus combinations of filters and lighting must be used to provide the details of the structures that are of interest. According to our experience best results are obtained for most specimens with a wavelength of 365–366 nanometers (UV-A). The optimum of filtering and exposure time has to be tested in a series of experiments [3]. The number and combination of filters varies greatly: exposure times may be between 10 seconds and 10 minutes, depending on the nature of the fossil material, the magnification and the intensity and incident angle of the ultraviolet lamps.

Recording the images work best with analogue (film) photography onto slide film although digital cameras can be used.

## Results

**Figure 2:**
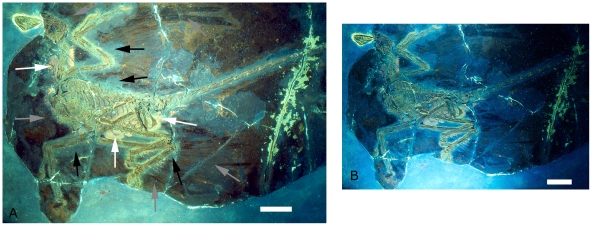
The holotype of *Microraptor gui*, IVPP V 13352 under UV light. Different filters were employed for parts A and B, hence the difference in colour and appearance. A also is labeled to indicate the preserved feathers (grey arrows) and the ‘halo’ around the specimen where they appear to be absent (black arrows) as well as phosphatised tissues (white arrows). Scale bars are 5 cm in both A and B.

Some of the resultant photographs are shown in figures 2–5. Numerous other slides were made and are archived at the IVPP.

**Figure 3 pone-0009223-g003:**
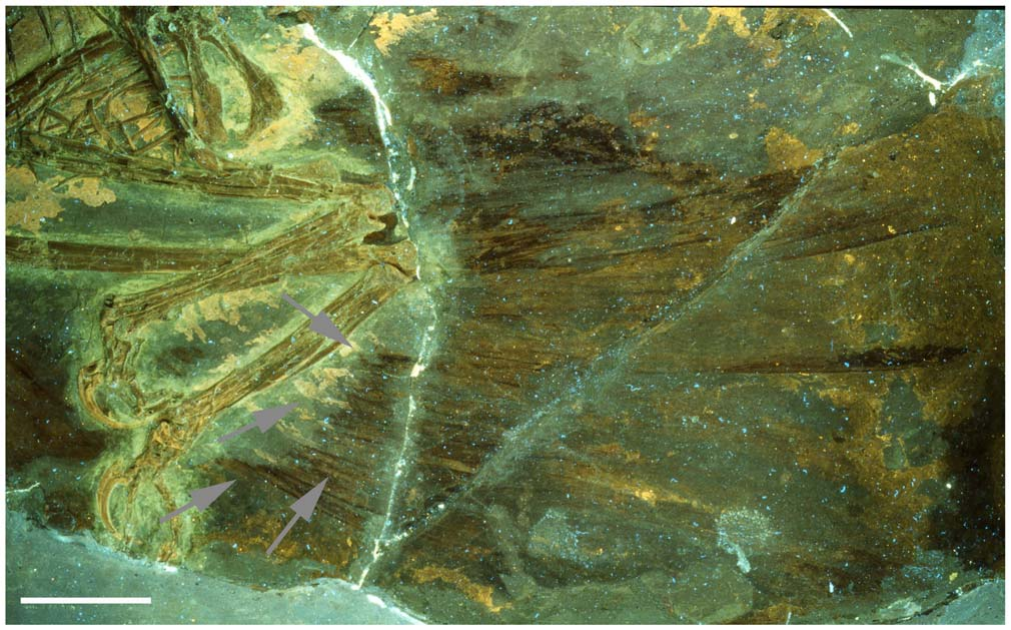
Close up of lower hindlimb of the holotype under UV light. This shows that the feathers do indeed penetrate the halo (grey arrows) when seen in UV and approach or reach the bones. These are not seen in natural light due to the overlying soft tissues seen in figure 2. Scale bar at 5 cm.

**Figure 4 pone-0009223-g004:**
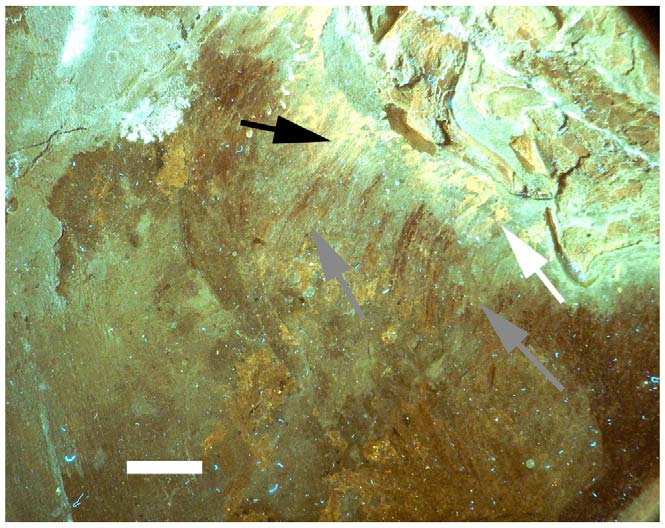
Close up of the chest of the holotype, close to the sternal plates under UV light. As with figure 3, this shows that the feathers do indeed penetrate the halo (grey arrows) when seen in UV and approach or reach the bones. These are not seen in natural light due to the overlying phosphatised tissues, but the striations of the feathers are clearly visible despite this covering. Scale bar of 1 cm.

**Figure 5:**
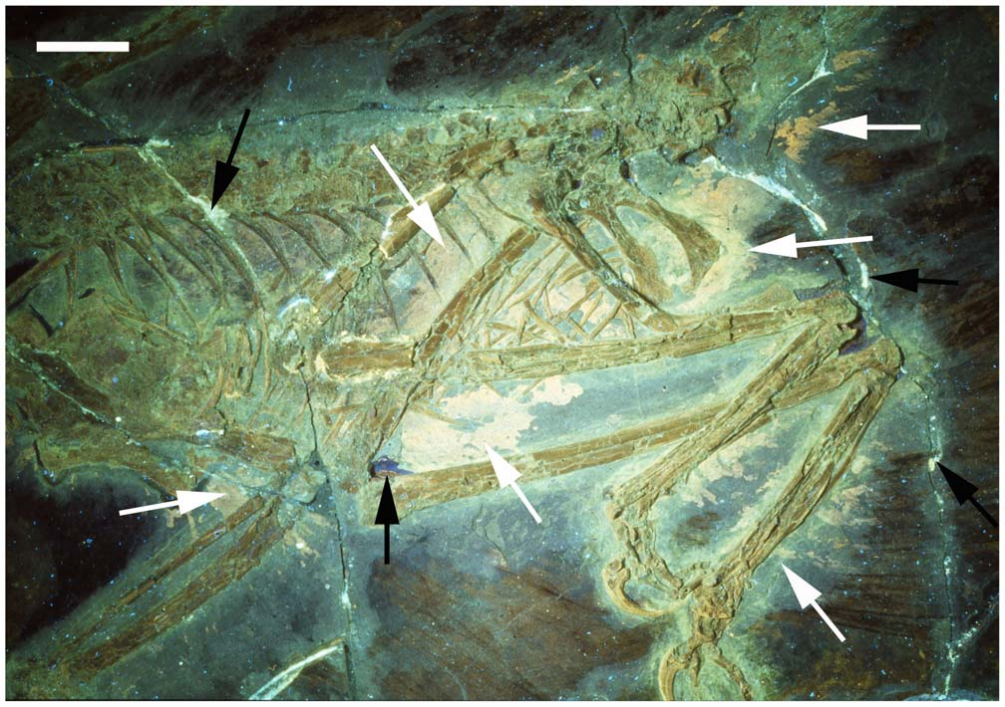
Close up of the lower part of the holotype under UV light. Variation in the phosphatised tissues can be seen (white arrows) as well as the bright reflectance of various glues and preservatives that have been applied to the specimen at various times (black arrows). Scale bar of 2 cm.

The use of UV light in addition to different filters during photography results in the various colours that are seen. These are caused by a combination of different absorption and reflectance of the various minerals that comprise the specimen and surrounding matrix or other factors. Occasional blue-white dots are dust motes that fluoresce brightly under intense UV and patches of glue/ solvents are clearly visible where parts of the slab were prepared (e.g. on the right hand side of figure 2). Figure 2 shows two different views of the specimen under different filter combinations, demonstrating the way in which some structures can fluoresce differently (or rather, be recorded differently) as a result. For example in 2B, the patch of soft tissue on the back of the neck is much duller than in 2A and can be seen more clearly as it contrasts better with the surrounding elements. In all cases, the matrix is somewhat dark. While there is variation in the colour of the matrix, the differences in tone and colour under UV are much less then in normal light, where the matrix is predominantly off-white but with darker and even black patches present.

Several features of the various tissues and especially feathers are visible under UV light, though the halo also remains visible. Under most filter regimes the bones show up as being pale green and are relatively uniform in colour and reflectance indicating homogeneous preservation of the bones (e.g. see figure 2). Large patches of indeterminate soft tissues are visible, notably around the neck, between the legs and around the pelvis and tail base (see figure 2). These can be identified as they fluoresce brightly and this suggests that they are phosphatised. Other patches of indeterminate body tissues are visible around the sternum and lower ribcage but are less bright and cannot always be seen with some filters (e.g. compare figure 2B with figure 5). Most soft tissues are associated with the skeleton but others are some distance from the bones.

The feathers are often hard to make out as they have a very low fluorescence/reflectance and thus are very dark. Longer exposures or the use of more powerful lights to boost contrast does not improve their visibility as light reflected from the bones and soft tissues can washout the image. Furthermore, in places the soft tissues overlap the feathers making identification of parts of them difficult (e.g. see figure 3). What is clear however, is that in places the feathers do extend into the halo around the bones (see figures 3 and 4 and below).

While the use of UV light does provide a better contrast between some elements of the specimen and others, it does not always reveal differences between elements of a certain kind. Thus, for example, identifying individual feathers from the mass of preserved feathers is no easier under UV than normal light.

## Discussion

Examination of the specimen under UV light and the resultant photographs demonstrate that the feathers of the *Microraptor gui* holotype extend into the ‘halo’ around the bones, in some cases as far as the bones themselves. In particular, this can be seen on the posterior face of the right metatarsus (see figure 3) and the furcula /sternal plate (figure 4). The former is especially important as it provides the confirmation of genuine attachment considered lacking by Padian and Dial [18]. In addition to this pattern being expected from how feathers articulate in modern birds (see above), this pattern matches that seen in other specimens from Liaoning, both in non-avian theropods and avians (e.g. *Beipiaosaurus* [34], *Epidexipteryx* [35] and *Eoconfuciusornis* [36]). This is also seen in *Archaeopteryx* from the Upper Jurassic Solnhofen deposits in Germany [see 1], [5]. As such we can be confident that this pattern is genuine and the feathers as preserved did extend to the bones of the specimen but are simply not visible under natural light.

The extension of the feathers into the halo and reaching the bones of the animal suggest strongly that these are in their natural position; i.e. they have not disarticulated and moved away from the body as a result of decomposition or water action. Indeed the lack of disturbance of non-primary and secondary feathers (such as those around the head and chest) indicate that the specimen was buried rapidly and suffered no great disturbance as these are normally the first to be lost during decomposition [37]. The preserved articulation of the feathers is therefore considered to be an accurate representation of their position in life. Some inferences can also be made about the orientation of the feathers, though as noted by Tischlinger and Unwin [5] care must be taken as the apparent position of the feathers in a fossil can be deceptive. Since the specimen is obviously compressed into two dimensions, the exact plane of the feathers with respect to the bones in 3D in life cannot be determined, but the relative positions of the feathers with respect to each other and the bones is likely correct.

It is clear that if the feathers have moved, they have not moved far. They retain contact with the bones of the specimen, none are significantly different from the position seen on modern birds (e.g. the feathers on the manus are subparallel to the fingers and the feathers are subparallel to each other), and none are articulated in places where they could not be present (e.g. on the unguals) or entirely separated from the skeleton. Thus, the general orientation of the feathers on the specimen is considered to be genuine, both with respect to the bones and to each other. The extension of the feathers into their halo also increases their known length, most notably with the body feathers and in places this would more double their apparent length (see table 1). Significantly, this also adds to the lengths of some long feathers of the manus and tibiotarsus as measured by both Xu and colleagues [11] and Chatterjee and Templin [19] and thus also would increase the wing areas as calculated in the latter publication.

**Table 1:** Change in feather lengths as a result of their extension into the halo. Measurements are taken along the curvature of the feather as far as possible. Only in some cases (marked with a *) can the feather be seen to reach the bone and thus its length measured. Other measurements are taken on the assumption that they do penetrate the halo and reach the bone (see text for details).

Feather type	Location	Apparent length (mm)	Full length (mm)
Plumulaceous	Back of the head	17	38
Plumulaceous	Base of the throat	8	23
Plumulaceous	Base of the spine	45	56
Plumulaceous	Chest (furcula)	14	23*
Plumulaceous	Proximal tail	13	24
Pennaceous	Distal right wing	105	113
Pennaceous	Middle of right wing	75	104
Pennaceous	Metatarsals (mid length)	105	113*
Pennaceous	Metatarsals (longest feather)	138	149

Even under UV light the feathers remain difficult to trace, largely as a result of the presence of phosphatised tissue that cover the area of the halo in places and also between the bones of the skeleton. There is no obvious structure or pattern to this material, and it is not clear what body tissues this originally was (i.e. muscles, interstitial tissue etc.). The halo itself may also be a result of a modification of the matrix owing to a reaction with the soft tissues. In places the feathers do show up as a carbonised stain on the rock that extends into the halo, but in other places are covered and only their structure and patterns are visible (see figure 4). This phosphatised tissue is inferred as part of the original body tissues of the animal that after death disassociated from the body and drifted or settled out (or even moved by diffusion) partly away from the bones before becoming fossilised. This leaves the tissue unstructured and patchy (e.g. see figure 5) rather than in a consistent mass on the bones as seen in at least some fossils (e.g. a specimen of the basal pterosaur *Anuroganthus* from the Solnhofen Lithographic Limestone, Germany, [cf. 38]). These phosphatised tissues therefore obscure the carbonised feathers and make them difficult to observe close to the bones. It is therefore likely that they simply lie on top of the feathers, and these are likely preserved but simply obscured in most places.

The apparent paradox that the feathers have remained in place while some of the soft tissues have moved is not in fact real. Among extant birds soft tissues can begin to decay almost instantly with bacteria colonizing the carcass of an animal within hours of death [37], while some feathers at least may stay articulated with the carcass for weeks [39]. As such it is reasonable to note that the feathers would retain a correct position and orientation on a dead dromaeosaur while body fluids and tissues decayed and covered some parts of the feathers. Notably the phosphatised tissues lie very close to the skeletal remains of the animal, which itself is in a natural posture. It seems unlikely therefore that any parts of the specimen were disturbed after coming to rest on the substrate after the initial death and possible transport.

Based on this new evidence we suggest that future research can assume that the feathers of *Microraptor gui* are preserved in a natural and correct position, though are somewhat longer than are visible under natural light. It is likely that other specimens from Liaoning similarly hide the details of feathers, or perhaps even other tissues, and care should be taken when evaluating material at face value. Certainly other specimens show a similar pattern with a featherless ‘halo’ (e.g. *Caudipteryx dongi*) or even where feathers run to the bones in some places but not others (e.g. *Longipteryx* [24]). While this is perhaps not a surprising conclusion based on how feathers (and especially long flight feathers) are attached to the body of birds, it is an important confirmation of this point. Furthermore, we would suggest that unless there is good reason to think that feathers have moved significantly away from a carcass, feathers should be assumed to be in a natural articulation and reach the bones, even where a halo is present. In short, the default hypothesis for analysing fossils such as *Microraptor* should be that feathers reached the bones and are preserved in articulation.

Further research into the taphonomy of these deposits is strongly encouraged to determine the processes at play and what other specimens may contain hidden material. We would also encourage curators, and especially preparators, to take care with exceptionally preserved specimens as they may contain hidden information that could be lost during preparation work if UV light is not employed. Just because specimens appear to lack soft tissues does not mean that feathers, skin and more might be lurking unseen in the matrix. Equally however, the liberal use of preservatives and glues can hide important details. Thus we warn against the practice of dousing whole (if fragile) specimens in consolidants that cannot be easily removed as these can mask such cryptic soft tissues as effectively as if they had been prepared off of the matrix.
